# The responses of pepper plants to nitrogen form and dissolved oxygen concentration of nutrient solution in hydroponics

**DOI:** 10.1186/s12870-024-04943-7

**Published:** 2024-04-13

**Authors:** Hamid Reza Roosta

**Affiliations:** Department of Horticultural Sciences, Faculty of Agriculture and Natural Resources, Arak University, Arak, Iran

**Keywords:** Ammonium, *Capsicum annuum*, Nitrate, Oxygen, Soilless culture

## Abstract

**Background:**

The presence of oxygen in the growth medium is absolutely essential for root development and the overall metabolic processes of plants. When plants do not have an adequate oxygen supply for respiration, they can experience a condition known as hypoxia. In order to investigate the impact of different nitrogen forms and varying oxygen levels in nutrient solutions on the growth, photosynthesis, and chlorophyll fluorescence parameters of bell pepper plants, a comprehensive study was conducted. The experiment was designed as a factorial experiment, considering two main factors: nitrogen forms (calcium nitrate and ammonium sulfate) with a fixed nitrogen concentration of 5 mM, and the oxygen levels of the nutrient solutions (ranging from 1.8 ± 0.2 to 5.3 ± 0.2 mg. L^-1^).

**Results:**

The study examined the effects of nitrogen (NH_4_^+^ and NO_3_^−^) application on various parameters of vegetative growth. The results demonstrated that the use of ammonium (NH_4_^+^) led to a reduction in the most measured parameters, including the fresh and dry mass of both the root and shoot, at low O_2_ concentrations of 1.8 ± 0.2; 2.6 ± 0.2 and 3.8 ± 0.2 mg. L^-1^. However, an interesting observation was made regarding the impact of oxygen levels on root growth in plants grown with nitrate (NO_3_^−^). Specifically, the highest levels of oxygen significantly increased root growth in NO_3_^−^-fed plants. Additionally, the application of NH_4_^+^ resulted in an increase in chlorophyll concentration in the leaves, particularly when combined with high oxygen levels in the nutrient solution. On the other hand, leaves of plants fed with NO_3_^−^ exhibited higher photosynthetic rate (A), intrinsic water use efficiency (iWUE), and instantaneous carboxylation efficiency (A/C_i_) compared to those fed with NH_4_^+^. Furthermore, it was found that NO_3_^−^-fed plants displayed the highest instantaneous carboxylation efficiency at oxygen levels of 3.8 and 5.3 mg. L^-1^, while the lowest efficiency was observed at oxygen levels of 1.8 and 2.6 mg. L^-1^. In contrast, NH_4_^+^-grown plants exhibited a higher maximal quantum yield of PSII photochemistry (F_v_/F_m_), as well as increased variable fluorescence (F_v_) and maximum fluorescence (F_m_), compared to NO_3_^−^-grown plants. Interestingly, the NO_3_^−^-fed plants showed an increase in F_v_/F_m_, F_v_, and F_m_ with the elevation of oxygen concentration in the nutrient solution up to 5.3 mg. L^-1^.

**Conclusion:**

This study showed that, the growth and photosynthesis parameters in bell pepper plants are sensitive to oxygen stress in floating hydroponic culture. Therefore, the oxygen level in the nutrient solution must not be lower than 3.8 and 5.3 mg. L^-1^ in NH_4_^+^ and NO_3_^−^ –supplied culture media or nutrient solutions, respectively.

## Background

Nitrogen (N) is a vital element for plant growth, and plants primarily acquire it through the uptake of NO_3_^−^ and NH_4_^+^ forms [1]. Nitrogen plays a crucial role as a constituent of many essential plant cell components, including amino acids and nucleic acids. However, it is widely recognized that high concentrations of NH_4_^+^ can have toxic effects on plants, leading to significant growth inhibition [2]. To understand the mechanisms underlying NH_4_^+^ toxicity, several hypotheses have been proposed [3]. These hypotheses primarily focus on the physiological changes associated with NH_4_^+^ assimilation, such as the uncoupling of photophosphorylation, as well as the disruption of ion balances due to reduced uptake of vital cations like potassium (K^+^), magnesium (Mg^2+^), and calcium (Ca^2+^) [3–5]. By investigating these hypotheses, researchers aim to shed light on the causes of NH_4_^+^ toxicity and develop strategies to mitigate its impact on plant growth and productivity. In many hydroponic systems, a combination of NO_3_^−^ and NH_4_^+^ sources of nitrogen is commonly utilized for better growth of the plants [4]. The enhancing impact of NH_4_^+^ supplementation in NO_3_^−^-fed plants is linked to decreased energy demands (3–12%) and changes in phytohormone equilibrium [6].

In higher plants, oxygen plays a crucial role as a limiting substrate for efficient energy production, necessitating metabolic adjustments based on oxygen availability. Despite their ability to produce oxygen in the presence of light, plants can encounter low oxygen conditions when oxygen diffusion from the environment fails to meet the demands set by metabolic rates [7]. This oxygen deficiency is particularly prevalent in certain hydroponic systems, notably floating culture [8]. Low concentrations of oxygen significantly impact root physiological functions, leading to decreased respiration and water uptake [9]. Furthermore, studies have reported that oxygen deprivation in the nutrient solution results in reduced nutrient levels in both the leaves and roots of tomato plants [10]. Morard et al. [11] demonstrated that oxygen deprivation leads to the accumulation of nitrite in the nutrient solution and xylem, with nitrite accumulation being one of the factors contributing to tissue damage under oxygen-deficient conditions. Additionally, their research revealed that diminished root activity resulting from oxygen deprivation leads to reduced absorption of water and essential nutrients such as potassium (K), magnesium (Mg), calcium (Ca), phosphorus (P), and sulfur (S). The lack of oxygen can also have a negative impact on plant metabolism, including nitrogen (N) uptake and assimilation [11]. Under oxygen-deficient conditions, the rates of NO_3_^−^ and NH_4_^+^ uptake are significantly reduced [12]. Consequently, plants grown in oxygen-deficient environments often exhibit symptoms of nitrogen deficiency alongside those associated with oxygen stress, as oxygen deficiency inhibits N uptake [13]. The impact of oxygen deficiency on various plant species has been extensively studied, revealing significant effects on critical physiological processes. For instance, flooding treatment has been observed to have a substantial influence on photosynthesis, antioxidant enzyme activity, shoot growth, and N absorption in rapeseed plants [14]. The growth of watermelon plants has also been found to decrease under oxygen-deficient conditions [15]. In another experiment, soybean plants exposed to oxygen deficiency and high carbon dioxide levels exhibited a reduction in the greenness index compared to plants grown under normal conditions. Furthermore, when the plant roots were exposed to 100% nitrogen gas, the chlorophyll content experienced the most significant decrease [16]. These findings highlight the detrimental consequences of oxygen deficiency on plant health and photosynthetic processes, emphasizing the importance of maintaining adequate oxygen levels for optimal growth and development.

Aeration plays a crucial role in closed hydroponic systems, but the specific conditions can vary across different systems. In the Nutrient Film Technique (NFT) system, as well as to some extent in Deep Flow Technique (DFT), the flow of nutrient solution through the plant cultivation channels promotes aeration and consequently enhances the level of dissolved oxygen. However, in these systems, plants with bulky roots can create anaerobic conditions around their roots by obstructing the flow of the nutrient solution. This issue becomes more pronounced in the floating culture system, where the absence of nutrient solution flow necessitates the introduction of air into the solution to ensure adequate aeration. Therefore, it is crucial to conduct research on the growth and physiology of plants under varying oxygen concentrations in the floating culture system, particularly when different nitrogen sources are present. This investigation will provide valuable insights into optimizing oxygen supply and nutrient delivery, ultimately leading to improved plant productivity and health in hydroponic systems.

Limited information currently exists regarding the correlation between inorganic nitrogen forms and dissolved oxygen levels, as well as their impact on pepper growth, photosynthesis, and chlorophyll fluorescence parameters. To address this knowledge gap, we conducted a hydroponic experiment wherein pepper plants were cultivated, and their growth, photosynthesis, and chlorophyll fluorescence parameters were measured. The study investigated the effects of four different concentrations of dissolved oxygen (1.8 ± 0.2; 2.6 ± 0.2; 3.8 ± 0.2; 5.3 ± 0.2 mg. L^-1^) and two forms of nitrogen nutrition (NH_4_^+^ and NO_3_^−^) on the aforementioned plant characteristics.

## Methodology

### Plant material and culture conditions

The experiment was conducted using pepper plants (*Capsicum annuum* cv. California Wonder) and followed a completely randomized design with three replications, employing a factorial combination. The first factor involved two forms of nitrogen (calcium nitrate and ammonium sulfate) at a concentration of 5 mM. The second factor consisted of four levels of dissolved oxygen in the nutrient solution: 1.8 ± 0.2; 2.6 ± 0.2; 3.8 ± 0.2; 5.3 ± 0.2 mg. L^-1^ O_2_.

The germination process began by sowing the pepper seeds in pots filled with a coarse (2–5 mm diameter) perlite medium. Once the seedlings reached the 4-true leaf stage (5 weeks after sowing the seeds), they were transplanted into individual buckets (16 cm diameter and 20 cm height) containing 4 L of aerated nutrient solution. Four plants were grown together in each bucket. Cultivation buckets were covered with lids and seedlings were placed in the holes built in the lid of the bucket so that there was a one-centimeter gap between the lid of the bucket and the nutrient solution, which was filled with air. The greenhouse’s reverse osmosis system, equipped with five filters, effectively supplied the necessary water. The outflow water from this system had an electrical conductivity (EC) of 14 µS.cm^-1^. The formula of the nutrient solution used in this experiment is shown in Table 1 [4]. . The greenhouse grade of chemicals was used to make the nutrient solution. The nutrient solution remained consistent across all treatments, except for the nitrogen form. The temperature of the nutrient solution remained at 22 ± 2 ºC during plant growth.

**Table 1:** Concentration of nutrients used in the nutrient solution of this experiment

Macronutrients	Concentration (mmol.L^-1^)	Micronutrients	Concentration (µmol.L^-1^)
KH_2_PO_4_	0.2	Fe(III)-EDTA-Na	20
K_2_SO_4_	0.2	H_3_BO_3_	2
MgSO_4_.7H_2_O	0.3	MnSO_4_.H_2_O	7
Ca(NO_3_)_2_.4H_2_O^*^	5	ZnCl_2_	0.7
(NH_4_)_2_SO_4_^*^	5	CuSO_4_.5H_2_O	0.8
NaCl	0.1	Na_2_MoO_4_.2H_2_O	0.8

^*^with either 5 mM nitrogen as calcium nitrate (Ca(NO_3_)_2_.4H_2_O) or ammonium sulfate ((NH_4_)_2_SO_4_)

During the cultivation period, the pH of the nutrient solution was maintained between 6 and 6.8 by utilizing 2.5 g/bucket calcium carbonate (CaCO_3_) as a buffer. The solutions were completely replaced every week for the initial five weeks, after which they were refreshed every fourth day [8]. The pepper plants were grown in a glass covered greenhouse located at Vali-e-Asr University, Rafsanjan, Iran, with an 11-hour photoperiod (26 ± 3 ºC) followed by a 13-hour dark period (23 ± 3 ºC), and 55% relative humidity. The temperature was controlled using an air conditioning system.

### Oxygen measurement and adjustment

During the treatment, an air pump with a power of 2 W was used to provide the necessary atmospheric O_2_. The air was distributed between the Buckets using a medical serum set by changing air follow into the nutrient solution. The oxygen levels were monitored daily and manually using a portable O_2_ meter (OXi 315, WTW Co., Germany) [8]. To achieve the desired O_2_ concentrations, a capsule containing nitrogen gas was used to inject N_2_ into the nutrient solution.

### Vegetative growth parameters

The plants were harvested 10 weeks after transplanting and their fresh mass was measured. The shoot and roots were then dried in an oven at 72 °C for 72 h and their dry mass was determined [8].

### Chlorophyll index

The SPAD-502 Chlorophyll Meter (Minolta Camera Co. Ltd., Osaka, Japan) was used to record the chlorophyll index in mature leaves. Three leaves were measured per plant, every 10 days.

### Leaf gas exchange

To evaluate plant leaf gas exchange parameters, a portable photosynthesis system (ADC BioScientific Ltd, Hoddesdon, UK) was utilized precisely 60 days after the initial planting. The parameters that were assessed included the net CO_2_ assimilation rate (A, µmol CO_2_ m^-2^.s^-1^), intrinsic water-use efficiency (WUEi, µmol CO_2_ mol^− 1^ H_2_O), stomatal conductance (G_s_, mol H_2_O m^-2^ s^-1^), transpiration rate (E, mmol H_2_O m^-2^.s^-1^), sub-stomatal CO_2_ concentration (C_i_, µmol CO_2_ mol^-1^), and instantaneous carboxylation efficiency (A/C_i_, mol CO_2_ m^-2^ s^-1^). These measurements were specifically conducted on fully expanded leaves between the hours of 9:00 AM and 1:00 PM.

### Chlorophyll fluorescence

The parameters were measured and calculated at the end of the growing period, specifically 60 days after planting. To carry out the measurements, a portable photosynthetic efficiency analyzer (PEA) manufactured by Hansatech Inc. Co., UK was used. The PEA allowed for the assessment of various parameters relevant to the photosynthetic activity of the plants.

The measured parameters included: Variable fluorescence (F_v_): This parameter indicates the difference between the minimal and maximal fluorescence levels emitted by the photosystem II (PSII) during light exposure. It provides insights into the efficiency of the light energy conversion process. Maximal fluorescence of the dark-adapted state (F_m_): Fm represents the highest possible fluorescence emission under dark-adapted conditions. It serves as a reference point for assessing the photosynthetic efficiency of the plant. Minimal fluorescence yield of the dark-adapted state (Fo): F_o_ signifies the minimal fluorescence emission under dark-adapted conditions. It aids in evaluating the efficiency of the energy transfer processes within the photosynthetic system. Maximal quantum yield of PSII photochemistry (F_v_/F_m_): This parameter is derived by dividing F_v_ by F_m_ and provides a measure of the maximum efficiency of the photosynthetic apparatus.

To conduct the measurements, fully expanded leaves were collected from each plant. Special tags were affixed to the upper leaf blades to ensure consistent positioning and to facilitate a dark adaptation period of 15 min before taking the measurements. Following the dark adaptation, a sensor cup was carefully placed on the leaf surface for the measurement process.

Chlorophyll fluorescence transients were induced by exposing the leaves to red light with an intensity of up to 3,500 µmol (photon) m^–2^ s^–1^. The fluorescence signals were recorded within a time range of 10 µs to 1 s, with the peak wavelength set at 627 nm. These measurements provide valuable information about the dynamic response of the photosynthetic system to light stimuli and help assess the overall photosynthetic performance of the plants. The fluorescence transients were analyzed according to the equations of the JIP-test [17].

### Statistical analysis

An analysis of variances was conducted utilizing SAS software (SAS Institute, Cary, NC) to enhance the statistical analysis. In the event that the treatments displayed significant effects (with a P-value of less than 0.05 according to the F-test), a Duncan’s test was employed to discern differences among the treatment means.

## Results and discussion

### Vegetative growth

**Fig. 1:**
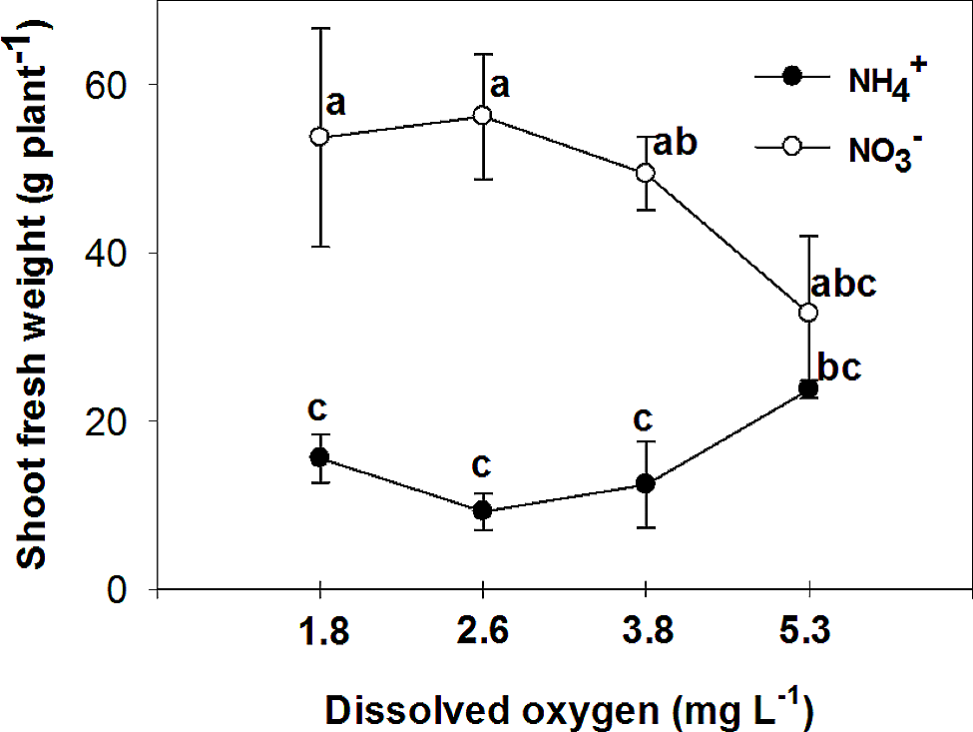
Effect of N-forms (at 5 mM) and dissolved O_2_ levels (1.8 ± 0.2; 2.6 ± 0.2; 3.8 ± 0.2; 5.3 ± 0.2 mg. L^− 1^) of nutrient solution on shoot fresh mass of bell pepper. Different letters show significant different, Duncan’s test (*p* ≤ 0.01)

**Fig. 2:**
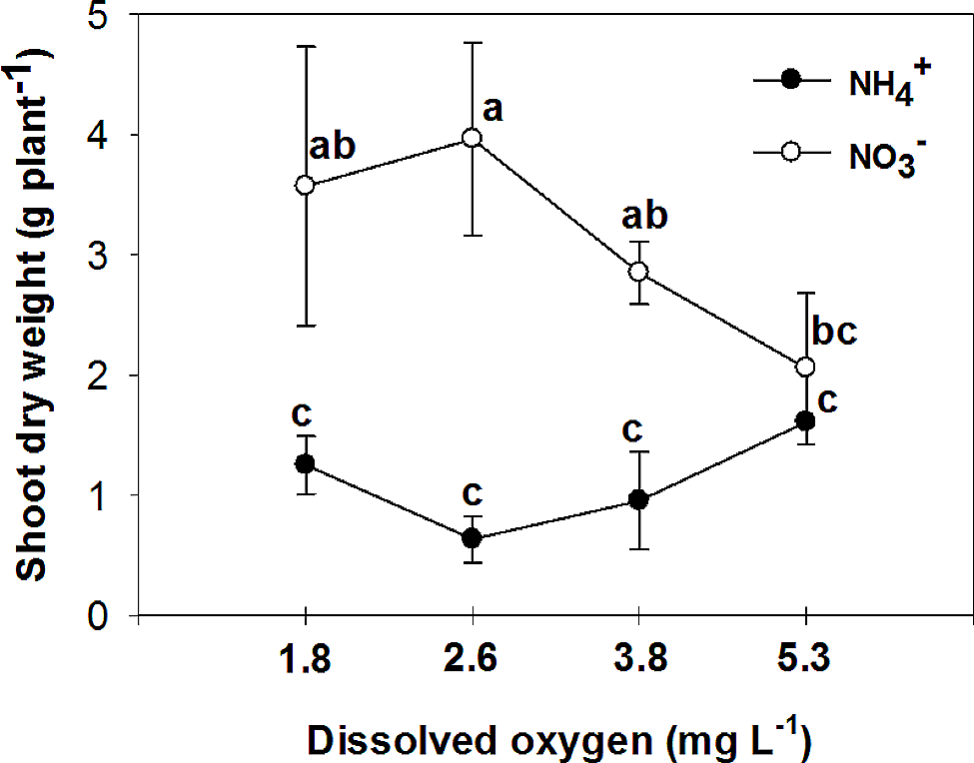
Effect of N-forms (at 5 mM) and dissolved O_2_ levels (1.8 ± 0.2; 2.6 ± 0.2; 3.8 ± 0.2; 5.3 ± 0.2 mg. L^− 1^) of nutrient solution on shoot dry mass of bell pepper. Different letters show significant different, Duncan’s test (*p* ≤ 0.01)

**Fig. 3:**
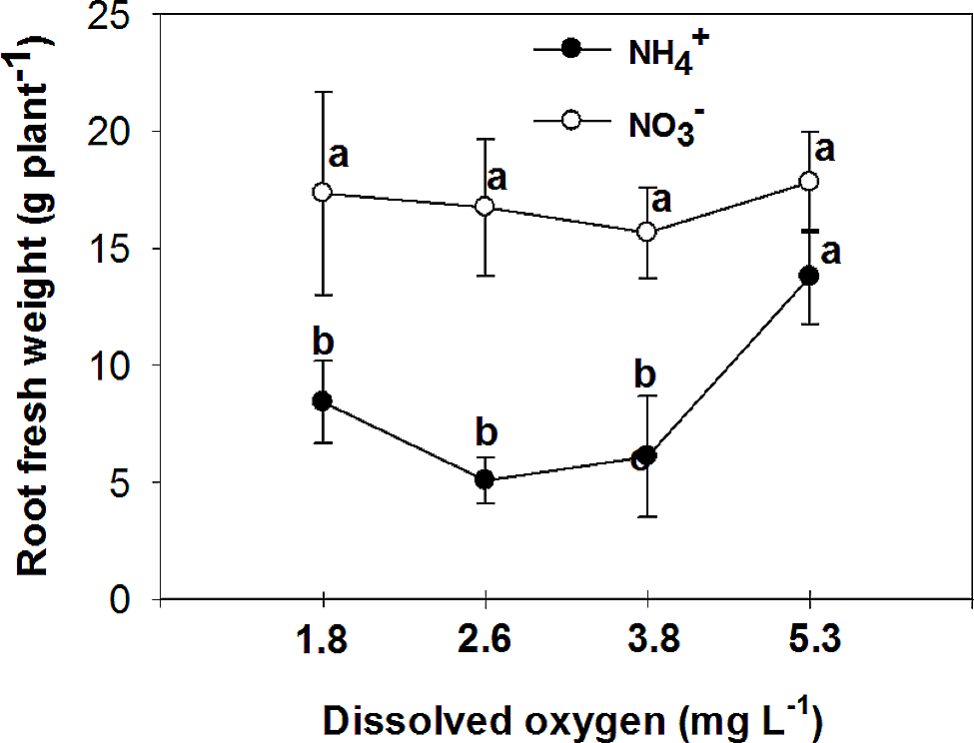
Effect of N-forms (at 5 mM) and dissolved O_2_ levels (1.8 ± 0.2; 2.6 ± 0.2; 3.8 ± 0.2; 5.3 ± 0.2 mg. L^− 1^) of nutrient solution on root fresh mass of bell pepper. Different letters show significant different, Duncan’s test (*p* ≤ 0.01)

**Fig. 4:**
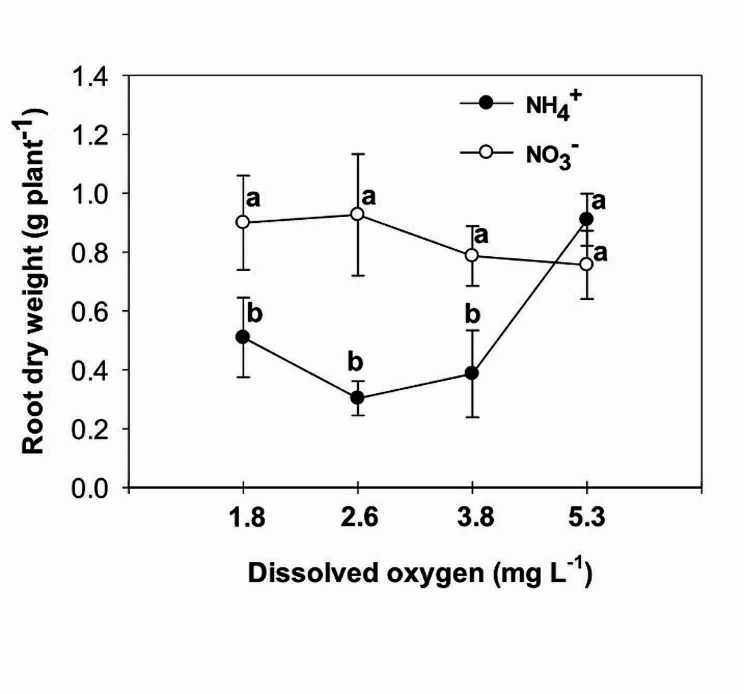
Effect of N-forms (at 5 mM) and dissolved O_2_ levels (1.8 ± 0.2; 2.6 ± 0.2; 3.8 ± 0.2; 5.3 ± 0.2 mg. L^− 1^) of nutrient solution on root dry mass of bell pepper. Different letters show significant different, Duncan’s test (*p* ≤ 0.01)

The study revealed a significant interactive effect between the form of nitrogen (N) and the concentration of oxygen (O_2_) in nutrient solutions on the fresh and dry mass of shoot and root. Plants that were fed with NO_3_^−^ exhibited higher fresh and dry mass of shoot and root in comparison to those fed with NH_4_^+^, at low O_2_ concentrations of 1.8 ± 0.2; 2.6 ± 0.2 and 3.8 ± 0.2 mg. L^-1^ (Figs. 1, 2, 3 and 4). Moreover, an increase in the concentration of O_2_ to 5.3 mg. L^-1^ resulted in a significant increase in root fresh and dry mass of NH_4_^+^ fed plants compared to lower O_2_ concentrations. As a result, the difference in root growth of plants fed with NO_3_^−^ and NH_4_^+^ was not significant at this O_2_ level (Figs. 3 and 4). However, there was no significant difference in shoot fresh mass of NH_4_^+^ grown plants at different O_2_ levels (Figs. 1 and 2). While there was a notable variance in the shoot fresh and dry mass of plants fed with NH_4_^+^ and NO_3_^−^ at three low concentrations of O_2_, no significant distinction was observed between the two nitrogen forms at the highest O_2_ concentration of 5.3 mg L^-1^ (Figs. 1 and 2).

Many studies have reported a decrease in biomass in plants that are fed with sole NH_4_^+^, including tomato [18], cucumber [19], lettuce [20], and onion [21]. This reduction in plant growth can be attributed to various factors, such as a decrease in nutrient uptake, hormonal imbalance, ethylene evolution, futile transmembrane NH_4_^+^ cycling, and carbon skeleton depletion in the root [2]. The reduction in photosynthesis [22] and leaf area [23] is also related to the reduction in plant growth caused by NH_4_^+^. On the other hand, an increase in biomass has been observed in cucumber [24], tomato [25], eggplant [8], and watermelon [26] when the O_2_ concentration is increased. It has been shown that tomato growth is inhibited at 33% of the ambient oxygen concentration (2.5-3 mg.L^-1^) in a hydroponic system [27]. The lack of oxygen in the root environment not only directly reduces the activity of the root, but also indirectly reduces the amount of photosynthesis, which in turn reduces the transfer of photosynthetic materials to the root, ultimately leading to a sharp reduction in root growth and destruction [28]. Biczak et al. [29] found a significant reduction in the maximum quantum yield of photosystem II under hypoxia conditions, especially in leaves at lower positions on the pepper plant. In the current experiment, consistent outcomes were noted in plants grown with NO_3_^−^, showing similar results. However, there was no discernible variance in the maximum quantum yield of photosystem II among pepper plants fed with NH_4_^+^ regardless of the varying levels of oxygen concentration. Oxygen deficiency in the nutrient solution can have a detrimental effect on photosynthesis in pepper plants. Without sufficient oxygen available in the root zone, the plant’s ability to uptake nutrients [30] and water is hindered, leading to decreased nutrient and water availability for the plant. This can disrupt the plant’s metabolic processes, including photosynthesis, as oxygen is necessary for energy production through the electron transport chain. As a result, photosynthetic activity is reduced [28], leading to decreased growth, lower yields, and overall poor plant health in pepper plants. Ensuring proper aeration and oxygen levels in the nutrient solution is crucial for optimizing photosynthesis and promoting healthy growth in pepper plants.

Waterlogging and reduced oxygen levels can negatively impact plant growth by decrease in chlorophyll production or its decomposition can lead to less intense photosynthesis and a lack of carbohydrates in the plant [30].

### SPAD index, photosynthesis, and chlorophyll fluorescence

**Fig. 5:**
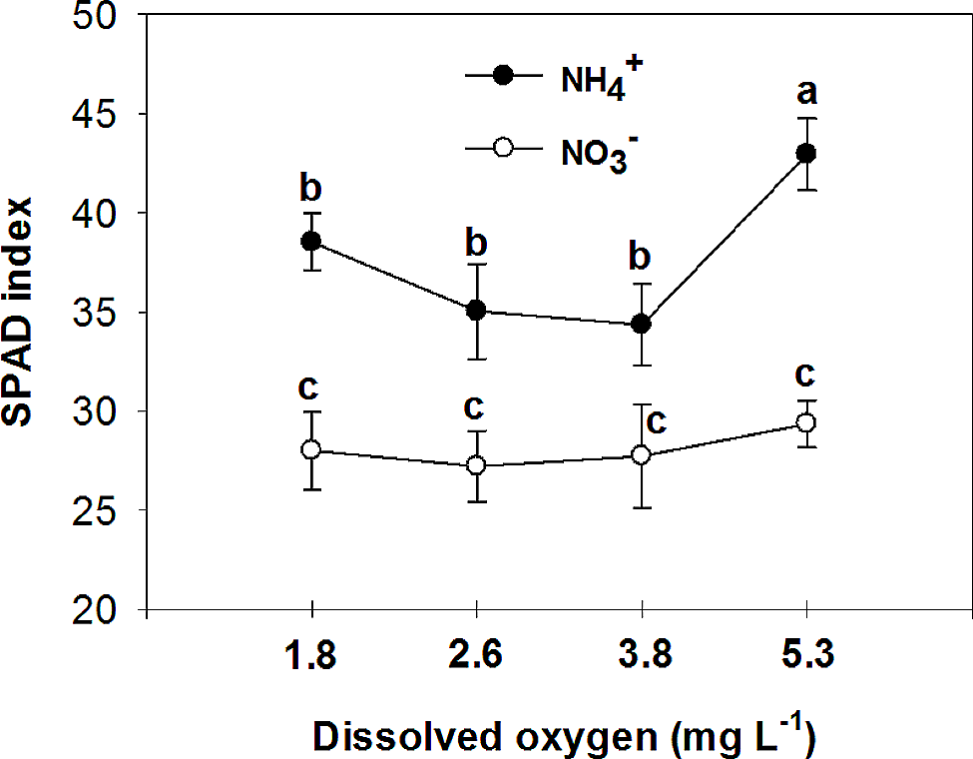
Effect of N-forms (at 5 mM) and dissolved O_2_ levels (1.8 ± 0.2; 2.6 ± 0.2; 3.8 ± 0.2; 5.3 ± 0.2 mg. L^− 1^) of nutrient solution on leaf SPAD index (leaf chlorophyll content) of bell pepper. Different letters show significant different, Duncan’s test (*p* ≤ 0.01)

The results showed that chlorophyll content (SPAD index) was higher in leaves of plants fed with NH_4_^+^ than those fed with NO_3_^−^ at all O_2_ concentration in nutrient solution (Fig. 5). The highest O_2_ level caused a significant increase in chlorophyll content in NH_4_^+^ fed plants, although different O_2_ levels in nutrient solution do not affect chlorophyll content of NO_3_^−^ grown plants. Increased chlorophyll content in NH_4_^+^-fed plants has been reported in cucumber [4, 19]. Ammonium has been found to have varying effects on chlorophyll concentration in plants. Studies have shown that mild (5 mM) ammonium concentration can lead to an increase in chlorophyll content in certain plant species, independent of their tolerance capacity [31]. However, exposure to higher concentrations of ammonium has been associated with a decrease in chlorophyll content in plants, leading to oxidative stress and changes in the activity of antioxidative enzymes [29, 32]. Therefore, the effect of ammonium on chlorophyll concentration in plants is dependent on the concentration of ammonium, and the specific plant species. The opposite results observed, where higher chlorophyll levels were found in NH_4_^+^-grown plants but with lower overall growth compared to nitrate-fed plants, can be explained as follows: The relationship between chlorophyll concentration and plant growth is intricate and can be influenced by various factors. For instance, studies have shown that NH_4_^+^ can enhance foliar color and increase chlorophyll concentration, but at the same time, it can negatively impact plant growth, as observed in cucumber [4] and tomato [19]. Similar results were observed with shading on *Kalmia latifolia* cultivars [33], while salt stress decreased chlorophyll concentration and inhibited the growth of maize plants [34]. In our previous experiment, we also found that NH_4_^+^-grown plants exhibited higher nitrogen (N) uptake compared to nitrate-fed plants [4]. Additionally, higher concentrations of total amino acids were observed in the NH_4_^+^-fed plants [4]. Therefore, the higher chlorophyll concentration in NH_4_^+^-grown plants could be attributed to the increased N absorption by these plants. However, the elevated N concentration resulting from NH_4_^+^ uptake can lead to nutrient imbalances within the plants [4]. Consequently, the relationship between chlorophyll concentration and plant growth is context-dependent, influenced by environmental conditions, and may vary across different plant species. In an experiment, it was observed that waterlogging (2 mg L^-1^ O_2_ concentration) had a significant impact on the amount of chlorophyll in leaves of tropical tolerant trees. While some species showed little change, others exhibited no difference in chlorophyll levels [35]. The hypoxia stress treatment significantly inhibited *Phyllostachys praecox* plant growth. Leaf chlorophyll contents was initially improved and then reduced with plant growth time [36]. The decrease in chlorophyll content can be attributed to several factors. One possible reason for the decrease in chlorophyll is the reduction in enzymes responsible for synthesizing photosynthetic pigments [37]. Under stress conditions, the activity of the chlorophyllase enzyme tends to increase [38], leading to a breakdown of chlorophyll. Additionally, the biosynthesis of new chlorophyll is hindered as stress conditions promote the synthesis of other compounds like proline. This shift in synthesis pathways reduces the availability of glutamate, a precursor needed for both chlorophyll and proline production. The variation in chlorophyll levels among plants under oxygen stress conditions can also be influenced by factors such as root structure and defense mechanisms. Some plants may develop misplaced roots [35] or employ other defense mechanisms to cope with waterlogging, which could affect chlorophyll content. Previous studies have reported significant decreases in fresh and dry mass in roots and stems, as well as chlorophyll content in corn plants exposed to waterlogged conditions [39]. These findings support the results of the current research. Waterlogging conditions can cause visible changes in leaf appearance and physiological characteristics include closing of stomata, reductions in the rate of photosynthesis and uptake of essential mineral nutrients, as well as alterations in plant growth hormones, often resulting in leaf discoloration and eventual yellowing [40]. This can be attributed to alterations in leaf structure and function caused by waterlogging. Furthermore, studies have shown that the concentration of chlorophyll decreases during oxygen deprivation [41]. Increased respiration has been observed in plants supplied with NH_4_^+^ [42], which requires higher oxygen levels. The assimilation of NH_4_^+^ necessitates an adequate oxygen supply for root cell respiration and the provision of carbon skeletons from the Krebs cycle. Higher oxygen levels in the nutrient solution may mitigate NH_4_^+^ toxicity by facilitating the detoxification of this ion through the provision of carbon skeletons for its incorporation into amino acids [43].

**Fig. 6:**
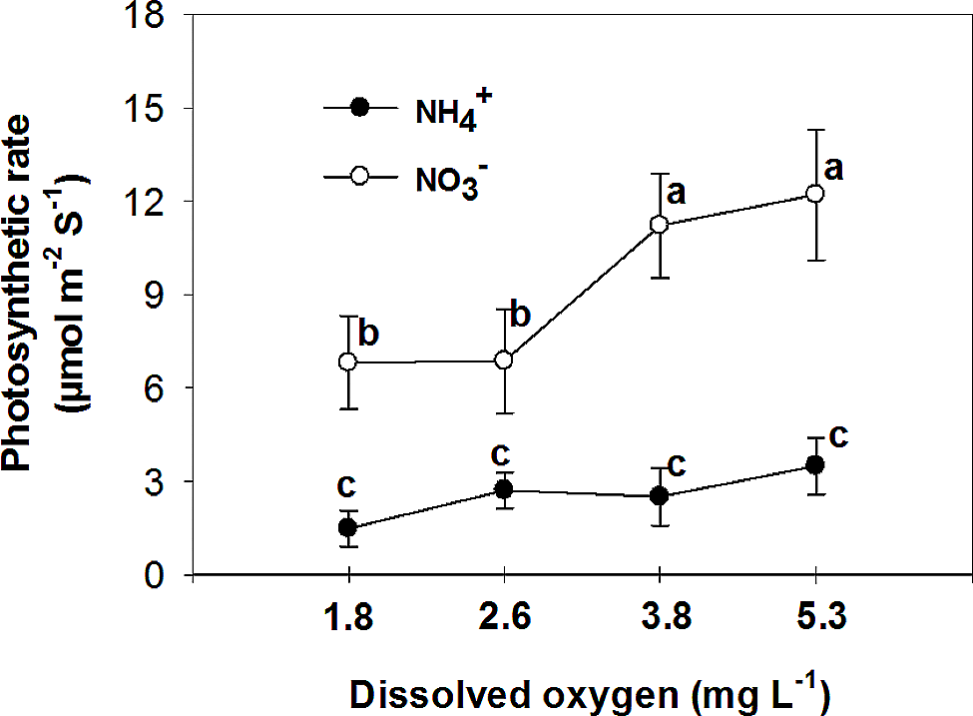
Effect of N-forms (at 5 mM) and dissolved O_2_ levels (1.8 ± 0.2; 2.6 ± 0.2; 3.8 ± 0.2; 5.3 ± 0.2 mg. L^− 1^) of nutrient solution on photosynthetic rate of bell pepper plants. Different letters show significant different, Duncan’s test (*p* ≤ 0.01)

**Fig. 7:**
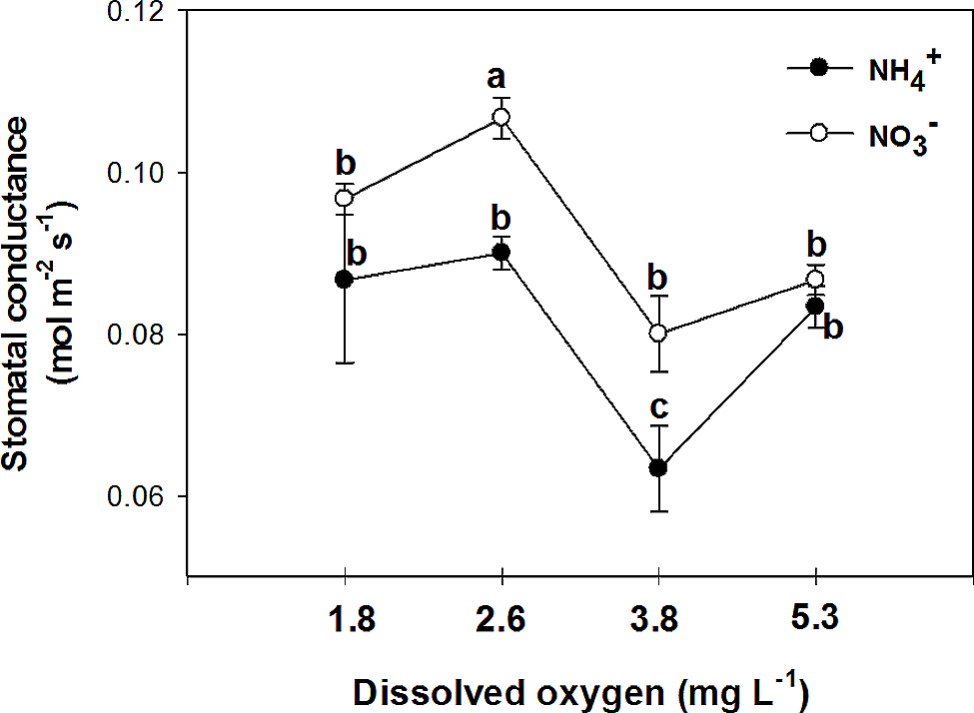
Effect of N-forms (at 5 mM) and dissolved O_2_ levels (1.8 ± 0.2; 2.6 ± 0.2; 3.8 ± 0.2; 5.3 ± 0.2 mg. L^− 1^) of nutrient solution on stomatal conductance of bell pepper plants. Different letters show significant different, Duncan’s test (*p* ≤ 0.01)

**Fig. 8:**
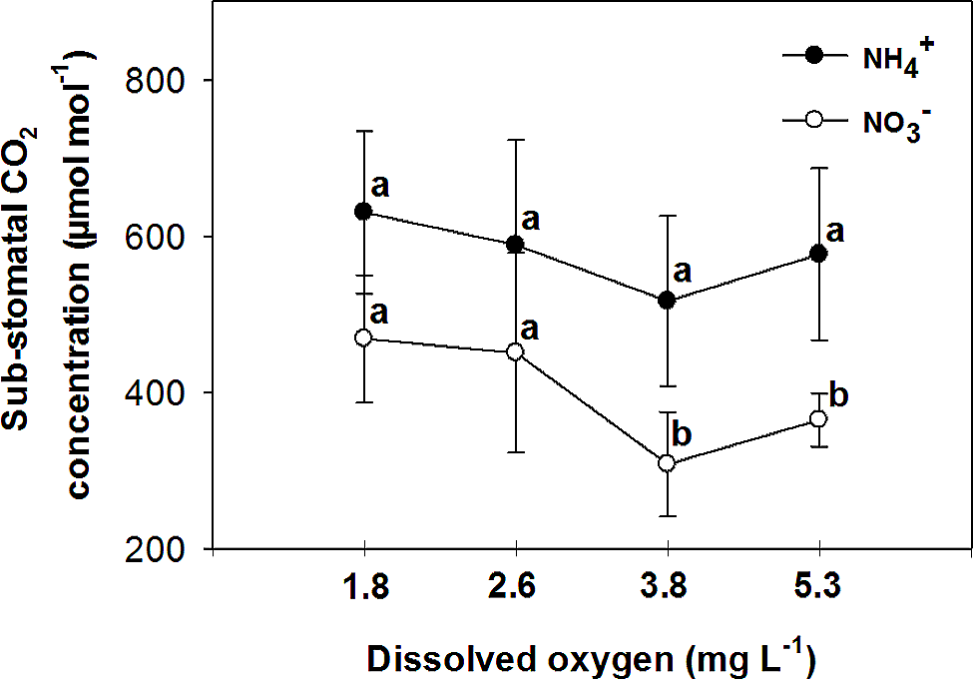
Effect of N-forms (at 5 mM) and dissolved O_2_ levels (1.8 ± 0.2; 2.6 ± 0.2; 3.8 ± 0.2; 5.3 ± 0.2 mg. L^− 1^) of nutrient solution on sub-stomatal CO_2_ concentration of bell pepper plants. Different letters show significant different, Duncan’s test (*p* ≤ 0.01)

**Fig. 9:**
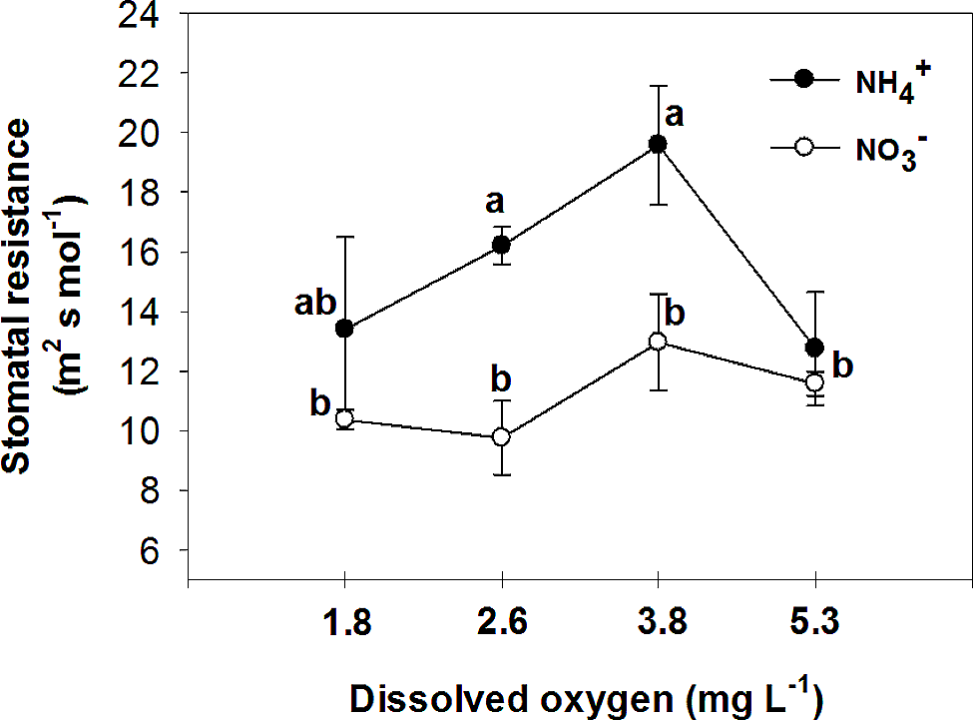
Effect of N-forms (at 5 mM) and dissolved O_2_ levels (1.8 ± 0.2; 2.6 ± 0.2; 3.8 ± 0.2; 5.3 ± 0.2 mg. L^− 1^) of nutrient solution on stomatal resistance of bell pepper plants. Different letters show significant different, Duncan’s test (*p* ≤ 0.01)

**Fig. 10:**
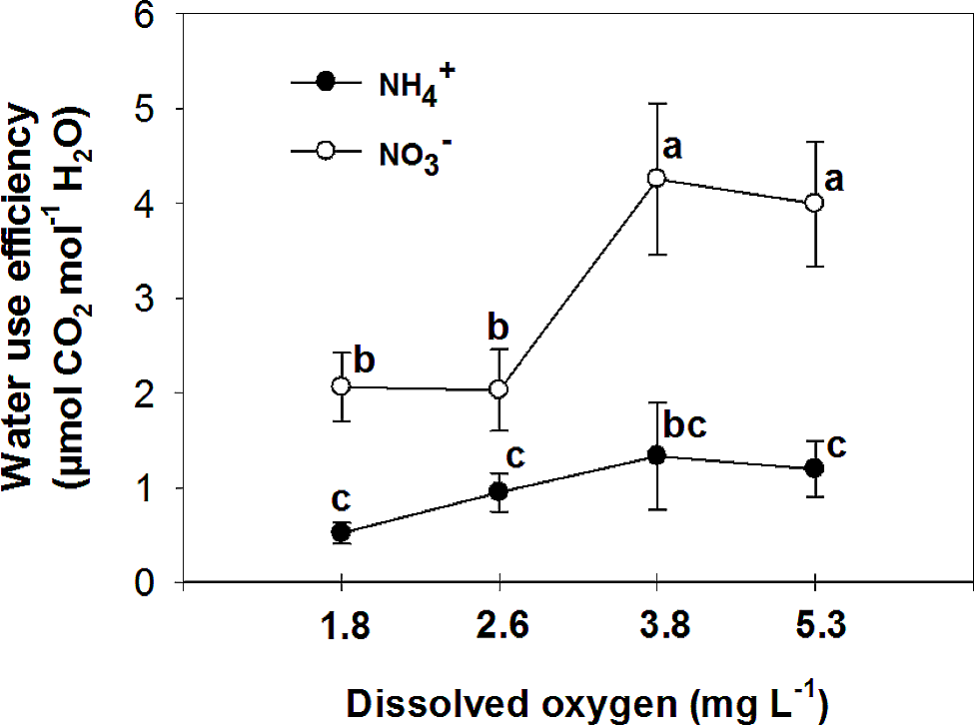
Effect of N-forms (at 5 mM) and dissolved O_2_ levels (1.8 ± 0.2; 2.6 ± 0.2; 3.8 ± 0.2; 5.3 ± 0.2 mg. L^− 1^) of nutrient solution on water use efficiency of bell pepper plants. Different letters show significant different, Duncan’s test (*p* ≤ 0.01)

**Fig. 11:**
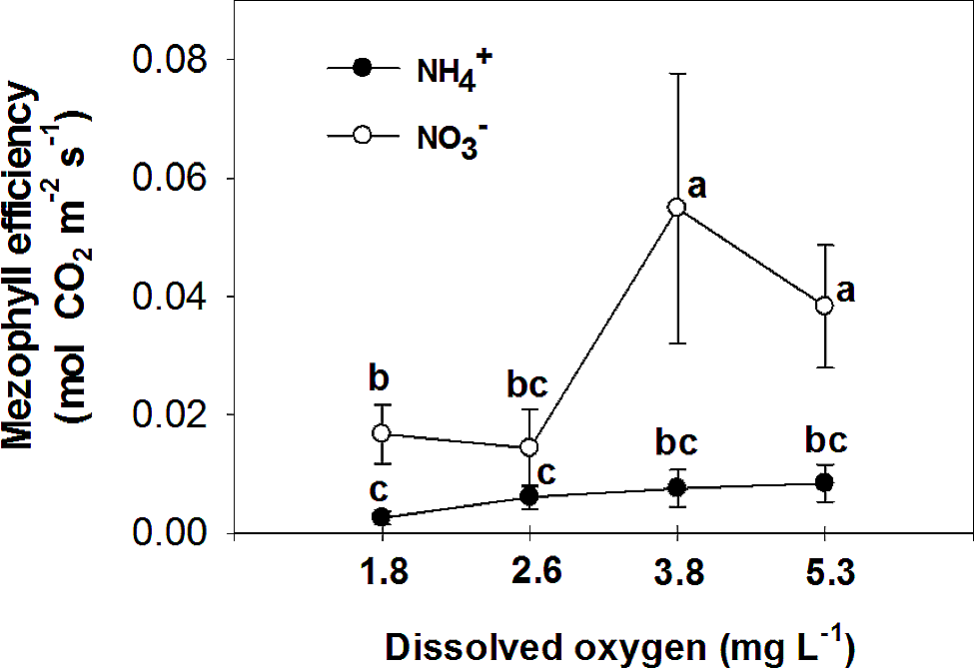
Effect of N-forms (at 5 mM) and dissolved O_2_ levels (1.8 ± 0.2; 2.6 ± 0.2; 3.8 ± 0.2; 5.3 ± 0.2 mg. L^− 1^) of nutrient solution on mesophyll efficiency of bell pepper plants. Different letters show significant different, Duncan’s test (*p* ≤ 0.01)

**Fig. 12:**
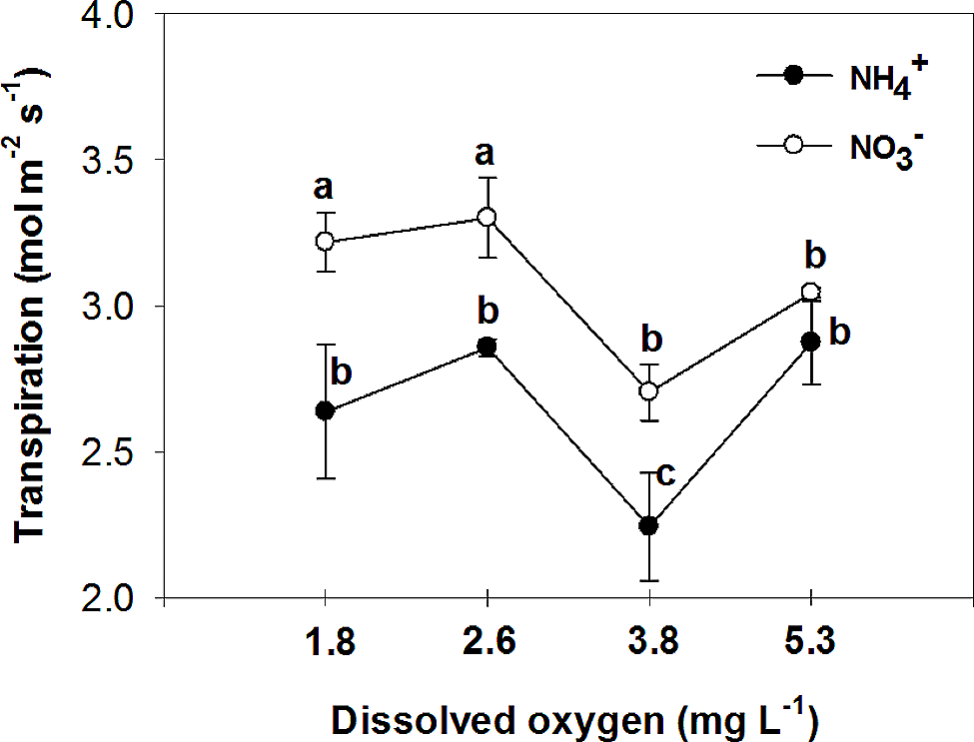
Effect of N-forms (at 5 mM) and dissolved O_2_ levels (1.8 ± 0.2; 2.6 ± 0.2; 3.8 ± 0.2; 5.3 ± 0.2 mg. L^− 1^) of nutrient solution on transpiration of bell pepper plants. Different letters show significant different, Duncan’s test (*p* ≤ 0.01)

Nitrate has been found to improve plant tolerance to oxygen deficiency, with foliar NO_3_^−^ assimilation being relevant to plant tolerance to oxygen deficiency [44]. Additionally, it has been observed that nitrogen application increases the plant’s tolerance to oxygen deficiency, with NO_3_^−^ treated plants showing higher CO_2_ assimilation and sucrose production compared to NH_4_^+^ treated plants under flooding conditions [45]. The current experiment demonstrated that the rate of photosynthesis was significantly higher in leaves of plants that were fed with NO_3_^−^ compared to those fed with NH_4_^+^ in all O_2_ concentration in nutrient solution (Fig. 6). Specifically, the plants fed with NO_3_^−^ exhibited the highest leaf photosynthetic rate at 3.8 and 5.3 mg. L^-1^ O_2_ levels, while the lowest rate was observed at O_2_ levels of 1.8 and 2.6 mg. L^-1^. These findings suggest that the detrimental impact of NH_4_^+^ supply on pepper growth may primarily be attributed to the inhibition of net photosynthesis activity. Previous studies by Board [46] and Lizaso et al. [41] have reported that a deficiency in O_2_ can reduce net photosynthesis. Furthermore, it was observed that leaves of plants fed with NO_3_^−^ had a higher stomatal conductance compared to those fed with NH_4_^+^ at O_2_ levels of 2.6 ± 0.2 and 3.8 ± 0.2 mg. L^-1^ (Fig. 7). Pepper plants exhibited the highest stomatal conductance with NO_3_^−^ nutrition at an O_2_ level of 2.6 mg. L^-1^ and the lowest with NH_4_^+^ nutrition at a level of 3.8 mg. L^-1^ O_2_. The nitrogen form did not affect stomatal conductance at the lowest and highest O_2_ concentrations in the nutrient solution. Additionally, the sub-stomatal CO_2_ concentration was found to be higher in leaves of NH_4_^+^-fed plants compared to those of NO_3_^−^-fed plants at 3.8 and 5.3 mg. L^-1^ O_2_ levels. As shown in the current experiment (Fig. 6), NH_4_^+^ nutrition can influence photosynthetic rates in plants. High levels of ammonium can inhibit photosynthesis, leading to a decrease in CO_2_ fixation. This reduction in CO_2_ assimilation can indirectly affect sub-stomatal CO_2_ concentrations. In contrast to NH_4_^+^-fed plants, NO_3_^−^-supplied plants generally exhibited a trend of decreasing sub-stomatal CO_2_ concentrations with increasing O_2_ levels in the nutrient solution (Fig. 8). These results can be related to the higher rate of photosynthesis in these treatments (Fig. 6). Stomatal resistance was higher in leaves of NH_4_^+^-fed plants compared to those of NO_3_^−^-fed plants, with the highest stomatal resistance observed at 3.8 mg. L^-1^ O_2_ in the nutrient solution for NH_4_^+^-grown plants (Fig. 9). This response is thought to be a mechanism by which plants regulate water loss, because, NH_4_^+^ restricts the water uptake in plants [47]. Unlike NH_4_^+^-fed plants, O_2_ levels had no effect on stomatal resistance in NO_3_^−^-supplied plants. Water use efficiency was higher in leaves of NO_3_^−^-fed plants compared to those of NH_4_^+^-fed plants at all O_2_ concentration in nutrient solution (Fig. 10). NO_3_^−^-fed plants exhibited the highest water use efficiency at 3.8 and 5.3 mg. L^-1^ O_2_ levels, while the lowest efficiency was observed at a level of 1.8 and 2.6 mg. L^-1^ O_2_. In NH_4_^+^-grown plants, different O_2_ levels in the nutrient solution had no effect on water use efficiency. In contrast of the current experiment, research has shown that NH_4_^+^-fed *Casuarina equisetifolia* plants exhibited higher water use efficiency and lower water consumption compared to plants supplied with NO_3_^–^, regardless of the water supply conditions [48]. This higher water use efficiency in NO_3_^–^ grown plants, as shown in the current experiment, was due to the higher rate of photosynthesis in these plants. The effect of oxygen deficiency in the nutrient solution on water use efficiency is a topic of interest in agricultural research. Studies have shown that oxygen deficiency in the nutrient solution can have immediate effects on the water uptake of plants [10]. Root asphyxia caused a decrease in water uptake [10]. Additionally, under root asphyxia conditions, plants may adapt to the new condition by relying on a metabolism of “nitrate respiration,” which could impact water and nitrate uptake processes, important factors for plant nutrition [10]. These findings highlight the importance of considering oxygen levels in the nutrient solution when aiming to optimize water use efficiency in plant cultivation. Moreover, the instantaneous carboxylation efficiency was higher in leaves of NO_3_^−^-fed plants compared to NH_4_^+^-fed plants (Fig. 11). Nitrate-fed plants demonstrated the highest instantaneous carboxylation efficiency at 3.8 and 5.3 mg. L^-1^ O_2_ levels, while the lowest efficiency was observed at a level of 1.8 and 2.6 mg. L^-1^ O_2_. Similar to water use efficiency, different O_2_ levels in the nutrient solution had no effect on the instantaneous carboxylation efficiency in NH_4_^+^-grown plants. The effect of ammonium nutrition on instantaneous carboxylation efficiency varies among plant species. Some studies have shown that ammonium nutrition can lead to a higher CO_2_ assimilation rate per leaf area compared to nitrate nutrition, indicating a potentially higher instantaneous carboxylation efficiency [49]. However, other research suggests that the growth of plants under high ammonium nutrition may be impaired, which could potentially affect carboxylation efficiency [50]. Additionally, the redox metabolism and mitochondrial electron transport chain play a role in the response to ammonium nutrition, which may also impact carboxylation efficiency [51]. Overall, the effect of ammonium nutrition on instantaneous carboxylation efficiency is complex and may depend on various factors such as plant species, growth conditions, and the specific mechanisms involved in ammonium assimilation and tolerance. Lastly, leaf transpiration was found to be the highest in NO_3_^−^-fed plants at the two initial concentrations of O_2_ (1.8 and 2.6 mg. L^-1^). The leaf transpiration of the other treatments remained at the same level (Fig. 12). The lowest leaf transpiration was observed in NH_4_^+^-fed plants at the 3.8 mg. L^-1^ O_2_. The effect of ammonium nutrition on transpiration is influenced by various factors, including the water lodging conditions. Research has shown that plants grown on ammonium nutrition exhibit lower water use efficiency compared to those receiving nitrate [52]. Additionally, the relationship between transpiration and soil water content suggests that once plants wilt, the transpiration rate should be roughly proportional to the available water content of the soil [53]. However, the specific interaction between ammonium nutrition and water lodging on transpiration requires further investigation.

**Fig. 13:**
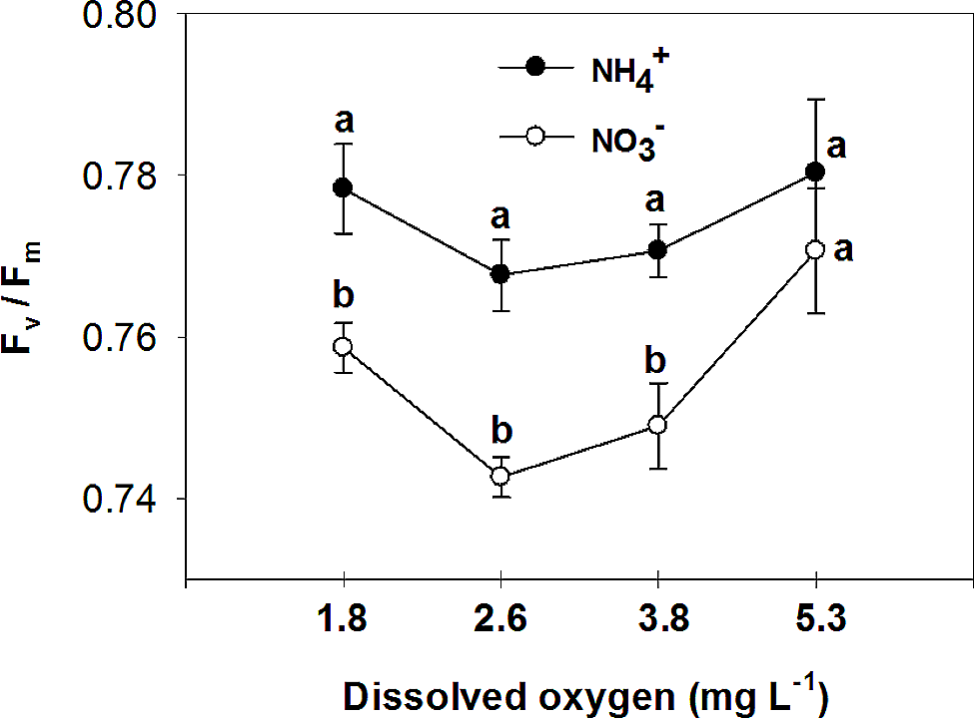
Effect of N-forms (at 5 mM) and dissolved O_2_ levels (1.8 ± 0.2; 2.6 ± 0.2; 3.8 ± 0.2; 5.3 ± 0.2 mg. L^− 1^) of nutrient solution on maximal quantum yield of PSII photochemistry (F_v_/F_m_) of bell pepper plants. Different letters show significant different, Duncan’s test (*p* ≤ 0.01)

**Fig. 14:**
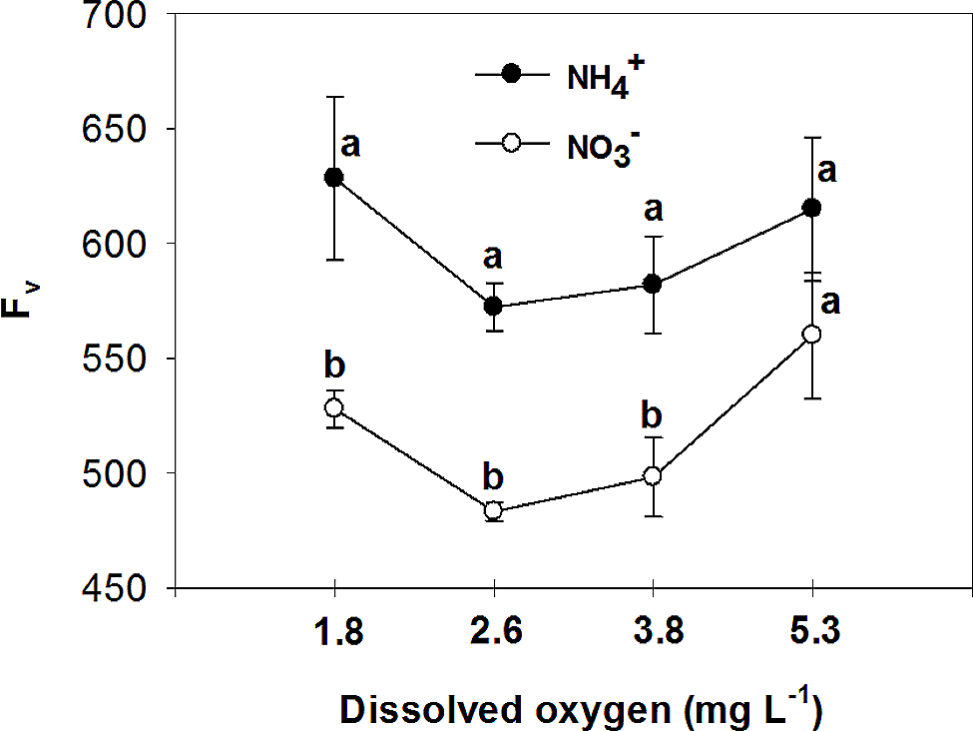
Effect of N-forms (at 5 mM) and dissolved O_2_ levels (1.8 ± 0.2; 2.6 ± 0.2; 3.8 ± 0.2; 5.3 ± 0.2 mg. L^− 1^) of nutrient solution on variable fluorescence (F_v_) of bell pepper plants. Different letters show significant different, Duncan’s test (*p* ≤ 0.01)

**Fig. 15:**
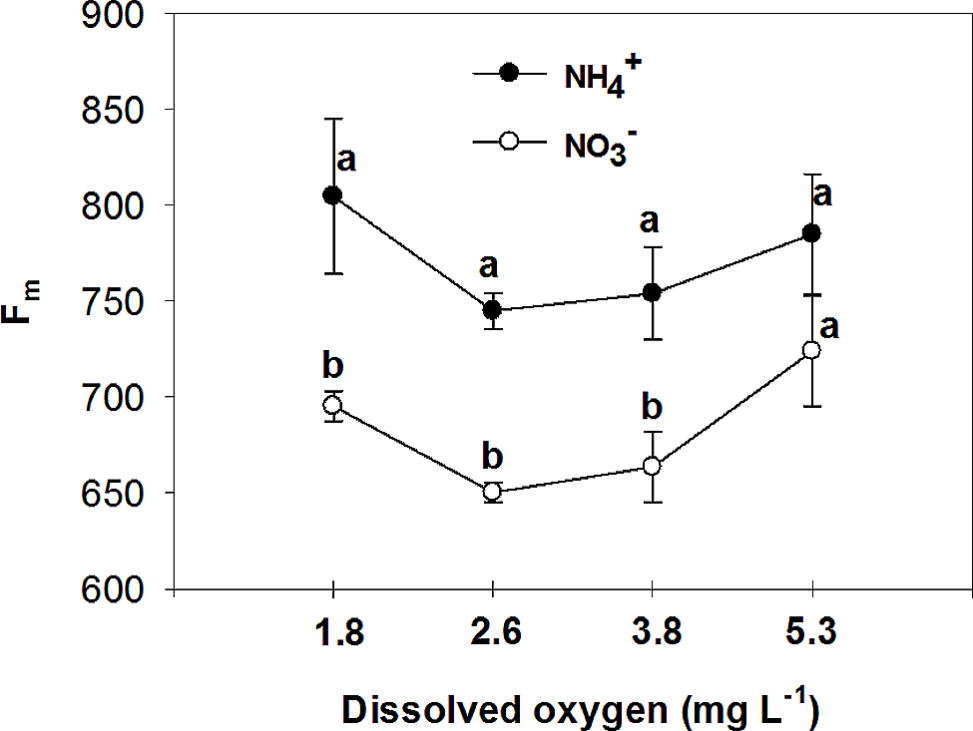
Effect of N-forms (at 5 mM) and dissolved O_2_ levels (1.8 ± 0.2; 2.6 ± 0.2; 3.8 ± 0.2; 5.3 ± 0.2 mg. L^− 1^) of nutrient solution on maximum fluorescence (F_m_) of bell pepper plants. Different letters show significant different, Duncan’s test (*p* ≤ 0.01)

**Fig. 16:**
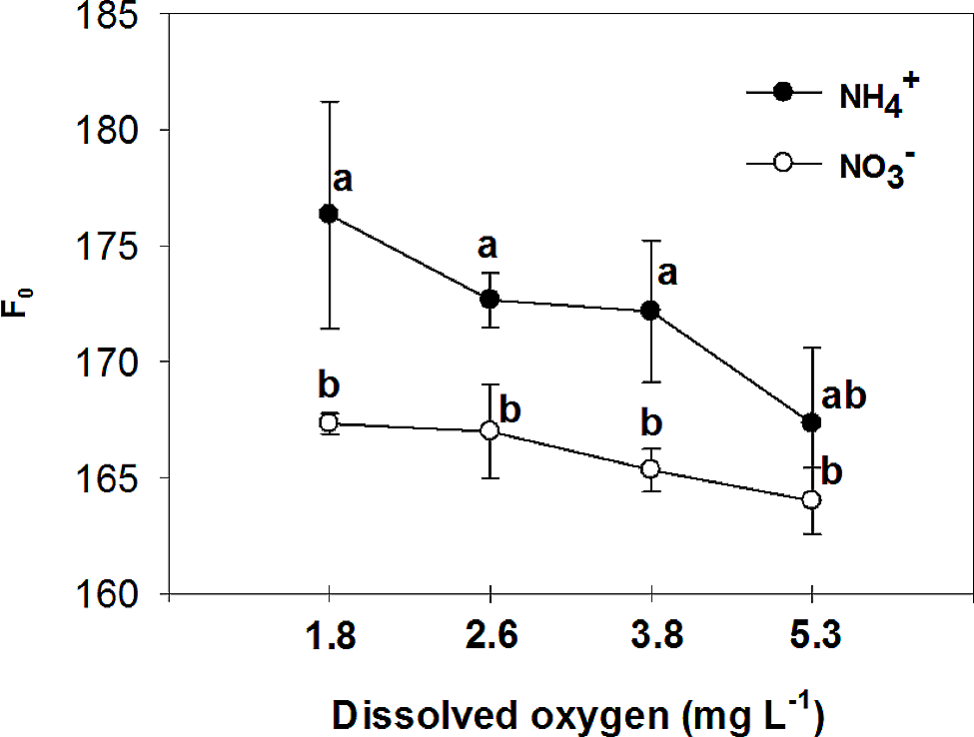
Effect of N-forms (at 5 mM) and dissolved O_2_ levels (1.8 ± 0.2; 2.6 ± 0.2; 3.8 ± 0.2; 5.3 ± 0.2 mg. L^− 1^) of nutrient solution on minimum fluorescence (F_o_) of bell pepper plants. Different letters show significant different, Duncan’s test (*p* ≤ 0.01)

The maximal quantum yield of PSII photochemistry, variable fluorescence, and maximum fluorescence were higher in NH_4_^+^ grown plants compared to NO_3_^−^ grown plants, but they increased in NO_3_^−^ -fed plants with the elevation of O_2_ concentration in the nutrient solution to 5.3 mg. L^-1^, without significant difference with NH_4_^+^ grown plants in the same O_2_ level (Figs. 13, 14 and 15). Minimum fluorescence also was higher in NH_4_^+^ grown plants compared to NO_3_^−^ grown plants, but it decreased with the elevation of O_2_ concentration in the nutrient solution to 5.3 mg. L^-1^ to the level of NO_3_^−^ grown plants, in the same O_2_ level (Fig. 16). In NO_3_^−^ grown plants, different O_2_ levels in nutrient solution had no effect on the minimum fluorescence of leaves. Ammonium nutrition has been shown to enhance chlorophyll fluorescence, specifically the F_v_/F_m_ parameter, in plants. A study on kohlrabi plants revealed that those given nitrogen as NH_4_^+^ showed a 21% increase in chlorophyll content, along with a reduction in the chlorophyll a: b ratio and decreased ground state fluorescence compared to plants supplied with nitrate [54]. In agree with the results of the current experiment, it was observed that the NH_4_^+^ -grown *Salvinia natans* plants exhibited a higher maximum quantum yield of PSII photochemistry (F_v_/F_m_) in hypoxic and anoxic conditions compared to NO_3_^−^ -fed plants [55]. However, Roosta et al. [19] reported that the form of nitrogen source does not affect the chlorophyll fluorescence of cucumber, which did not agree with the results of this research. The high nonphotochemical quenching shown in tomatoes fed with NH_4_^+^ or urea indicated that PS II was the inhibitory site of NH_4_^+^-N which was directly uptaken by roots, or liberated via the urea hydrolysis cycle. However, in the current experiment opposite results were observed, which may be due to the lower concentration of NH_4_^+^ in nutrient solution, and higher chlorophyll content in the NH_4_^+^-fed plants compared to the NO_3_^−^-fed plants [56]. Similar to the present study, Roosta and Schjoerring [4] found that NH_4_^+^ nutrition at the medium concentration (5mM) compared to NO_3_^−^ nutrition increased the chlorophyll concentration in cucumber leaves. Therefore, NH_4_^+^ does not affect the reaction center of photosystem II at 5 mM because of several protective mechanisms. If the carbon dioxide supply becomes limiting due to decreasing stomatal conductance as it was shown in current experiment, photorespiration acts as an alternative electron sink for the light reaction [57]. The latter protects PSII from damage during NH_4_^+^ stress, and therefore, F_v_/F_m_ is not a good indicator for detecting plant NH_4_^+^ response at mild NH_4_^+^ stress [58].

Hypoxic stress has been found to significantly impact the chlorophyll fluorescence parameter F_v_/F_m_, which serves as a sensitive indicator of environmental stress in plants [59]. A study conducted on cucumber plants subjected to hypoxia treatment demonstrated a decrease in F_v_/F_m_, indicating the occurrence of photoinhibition in photosynthesis [60]. Interestingly, this outcome aligns with the results of the current experiment involving plants supplied with nitrate. These findings collectively underscore the detrimental effects of hypoxic stress on chlorophyll fluorescence and photosynthetic activity in plants.

## Conclusions

This study has found that the overall growth of pepper plants was significantly reduced by NH_4_^+^ at low O_2_ concentrations in nutrient solution. However, the highest levels of oxygen increased vegetative growth, particularly root growth in NH_4_^+^ fed plants. The results also showed that chlorophyll content was higher in leaves of plants fed with NH_4_^+^ than in those fed with NO_3_^−^ at low O_2_ concentrations in nutrient solution. The highest O_2_ level caused a significant increase in chlorophyll content in NH_4_^+^ fed plants. Photosynthetic rate, water use efficiency, and instantaneous carboxylation efficiency were all higher in the leaves of NO_3_^−^ -fed plants compared to those of NH_4_^+^-fed plants at all O_2_ levels. Nitrate-fed plants had the highest instantaneous carboxylation efficiency at 3.8 and 5.3 mg. L^-1^ O_2_ levels and the lowest at levels of 1.8 and 2.6 mg. L^-1^ O_2_. Maximal quantum yield of PSII photochemistry, variable fluorescence, and maximum fluorescence were higher in NH_4_^+^ grown plants compared to NO_3_^−^ grown plants, but they increased in NO_3_^−^ -fed plants with the elevation of O_2_ concentration in the nutrient solution to 5.3 mg. L^-1^, without significant difference with NH_4_^+^ grown plants in the same O_2_ level. Therefore, to grow healthy pepper plants in a floating hydroponic system, it is important to control the O_2_ level and ensure it is not lower than 5.3 mg. L^-1^.

## Data Availability

All the data generated or analyzed during the current study were included in the manuscript. The raw data is available from the corresponding author on reasonable request.
